# Genomic and mutational landscape of anaplastic ependymoma: Insights from the AACR Project GENIE Consortium

**DOI:** 10.17305/bb.2026.13602

**Published:** 2026-04-21

**Authors:** Edie Gobel, Grace S Saglimbeni, Iosef I Perez, Bhanu Surabi Upadhyayula, Tyson J Morris, Akaash Surendra, Beau Hsia, Abubakar Tauseef

**Affiliations:** 1 School of Life Sciences, Arizona State University, Tempe, AZ, USA; 2 School of Medicine, Creighton University, Phoenix, AZ, USA; 3 School of Biological Sciences, University of California, Davis, CA, USA; 4 School of Medicine, Creighton University, Omaha, NE, USA

**Keywords:** Ependymoma, anaplastic ependymoma, AACR project GENIE, somatic mutations, *TERT, p53, NF2, NOTCH*, biomarker discovery, cancer genomics

## Abstract

Anaplastic ependymoma (AE) is a rare and aggressive central nervous system tumor that predominantly affects children and remains inadequately characterized at the genomic level. This study aimed to delineate the genomic and demographic landscape of histologically defined AE while identifying potential therapeutic targets. We conducted a retrospective analysis of AE cases from the American Association for Cancer Research (AACR) Project Genomics Evidence Neoplasia Information Exchange (GENIE) repository via cBioPortal, examining recurrent somatic mutations, copy number alterations, mutation co-occurrence, and exploratory sex- and race-based enrichment using descriptive and non-parametric statistics. The most frequent alterations included mutations in the telomerase reverse transcriptase (*TERT*) promoter, followed by recurrent changes in lysine methyltransferase 2D (*KMT2D*), lysine methyltransferase 2A (*KMT2A*), lysine methyltransferase 2C (*KMT2C*), E1A binding protein p300 (*EP300*), additional sex combs like 1 (*ASXL1*), and SET domain containing 2 (*SETD2*), indicating significant disruption of chromatin remodeling. Recurrent alterations in tumor protein p53 (*TP53*), ataxia telangiectasia mutated (*ATM*), and cyclin-dependent kinase inhibitor 2A (*CDKN2A*) suggested dysregulation of the p53 and DNA damage response pathways. Additionally, alterations in notch receptor 1 (*NOTCH1*) and notch receptor 2 (*NOTCH2*) indicated aberrant NOTCH signaling. Neurofibromin 2 (*NF2*) mutations were observed in male patients, and exploratory subgroup differences emerged across racial groups. Overall, AE appears to be driven by recurrent alterations in chromatin remodeling, p53, DNA damage response, and NOTCH signaling pathways, highlighting these areas as priorities for future biological validation and therapeutic investigation.

## Introduction

Anaplastic ependymoma (AE) is a rare and aggressive cancer of the central nervous system (CNS) that originates from ependymal cells and is classified as a neuroepithelial tumor [1]. The World Health Organization (WHO) categorizes this cancer as a Grade III malignancy [1]. Histopathologically, ependymomas demonstrate considerable variation and are typically classified into epithelial or glial subtypes [2]. In children, ependymomas primarily develop in the brain, while in adults, they are more frequently found in the spinal cord [1]. Clinical manifestations may include intracranial pain, personality changes, seizures, and focal neurological deficits [3].

The incidence of AE peaks in individuals aged 0–4 years [2]. Evaluation of suspected AE generally commences with contrast-enhanced Magnetic Resonance Imaging (MRI), which is the preferred imaging modality. Computed Tomography (CT) scans may also be valuable for identifying calcifications [1], an important diagnostic characteristic of AE. Treatment strategies depend on tumor location, stage, and metastatic potential, typically beginning with surgical resection followed by radiation therapy [4]. Chemotherapy may also be considered [3]. If complete tumor resection is not achieved, recurrence is likely, leading to disease progression. To enhance patient outcomes for AE, novel therapeutic approaches targeting fibroblast growth factor receptors are currently under investigation [6].

Previous research indicates that although AEs may appear identical microscopically, they exhibit genetic variations [1]. These tumors can arise in various CNS locations, with distinct molecular drivers that vary by site. Supratentorial ependymomas, affecting the upper brain region, are often driven by zinc finger translocation associated (*ZFTA)* fusion proteins, as well as Yes1 associated transcriptional regulator (*YAP1)* and cyclin dependent kinase inhibitor 2A (*CDKN2A)* deletions [7]. Tumors located in the posterior fossa, at the base of the skull, are commonly driven by EZH inhibitory protein (*EZHIP)* overexpression and gain of 1q [7]. Spinal ependymomas are primarily driven by MYCN proto-oncogene, bHLH transcription factor (*MYCN)* amplification and neurofibromin 2 (*NF2)* mutations [7].

While the precise etiology of AE remains elusive, significant strides have been made in understanding its risk factors and underlying drivers. However, a comprehensive understanding of the genetic alterations associated with AE is still lacking. Investigating these genetic variations, along with treatment resistance and disease progression, may lead to the identification of innovative interventions for AE. This study utilizes available cohort data to delineate the landscape of AE, with the aim of informing future interventions and screening methodologies.

It is essential to contextualize this study within the evolving framework of CNS tumor classification. The cohort analyzed herein was defined by the historical histopathological diagnosis of “anaplastic ependymoma” as recorded in the American Association for Cancer Research (AACR) Project Genomics Evidence Neoplasia Information Exchange (GENIE) database. This designation predates the 2021 5th Edition of the WHO Classification of CNS Tumors, which has largely supplanted histological grading with an integrated diagnosis based on molecular characteristics (e.g., *ZFTA* fusions) and anatomical location [7]. Due to the lack of necessary methylation or fusion data in the GENIE database, reclassification of this cohort according to contemporary WHO criteria was not feasible. Consequently, our findings reflect the genomic landscape of a histologically defined group and should be interpreted as a valuable dataset bridging the gap between historical classifications and the current molecular era.

## Materials and methods

### Data source and cohort selection

We retrospectively queried the AACR Project GENIE Consortium database (v18.0-public) on July 27, 2025, via cBioPortal for Cancer Genomics [8]. The patient cohort was defined by selecting those diagnosed with “Ependymoma” and a histology of “Anaplastic Ependymoma,” resulting in a cohort of 136 samples from 118 unique patients. Clinical outcome data, including survival information, was unavailable; thus, survival analysis could not be conducted.

### Genomic data processing and analysis

Comprehensive mutation analyses of tumor biopsies are widely employed across cancer types to characterize genomic alterations and inform disease biology, including in hematologic malignancies [5]. The AACR GENIE genomic database aggregates data from whole-exome sequencing (WES), whole-genome sequencing (WGS), and targeted panel sequencing (50–555 total genes) across nineteen international cancer centers. Participating institutions utilize specific pathways for mutation calling and annotation; however, adherence to GENIE protocols aligns with Genome NEXUS standards. For this analysis, somatic mutations were filtered to include only nonsynonymous variants (missense, nonsense, frameshift, splice-site) with a variant allele frequency (VAF) ≥5% and sequencing coverage ≥100×. Copy number alterations (CNAs), including homozygous deletions and amplifications, were assessed using thresholds identified by cBioPortal’s “CNA Genes” summary table. Although samples were generally obtained prior to treatment for molecular analysis, detailed longitudinal treatment data were not available for the AE cohort. The somatic mutation frequencies presented in the Results section were obtained from the “Mutated Genes” table in cBioPortal, encompassing both coding variants and curated non-coding alterations, such as telomerase reverse transcriptase (TERT) promoter mutations. These frequencies may differ from those shown in the OncoPrint, which represents a combination of mutation and copy-number events.

The cohort included 136 tumor samples from 118 unique patients. For demographic comparisons, including race and ethnicity regarding gene alterations, analysis was performed at the patient level, meaning that alterations were considered present if identified in any sample corresponding to a given patient. For sex- and race-based subgroup analyses, patients with unknown or unreported demographic annotations were excluded prior to analysis to ensure comparisons were restricted to patients with relevant annotations. *P* values were calculated using two-sided Fisher’s exact tests due to small subgroup sizes.

Percentages reported in demographic enrichment analyses reflect the proportion of patients within each demographic subgroup who were profiled for and altered in a given gene, as defined by cBioPortal, rather than the total subgroup counts shown in Table 1. As sequencing panel composition varied across contributing institutions, the number of patients profiled for each gene differed by subgroup, resulting in gene-specific denominators. Because GENIE v18 is no longer available in cBioPortal and gene-level ’profiled’ counts by subgroup were not retained during the original analysis, the per-gene subgroup denominators (N) cannot be retrospectively reconstructed for all comparisons.

**Table 1:** Patient demographic and tumor-sample characteristics

**Demographics**	**Category**	***n* (%)**
Sex	Male	53 (44.9%)
	Female	63 (53.4%)
	Unknown/not collected	2 (1.7%)
Age category	Adult	34 (28.81%)
	Pediatric	84 (71.19%)
Ethnicity (self-reported)	Non-hispanic	62 (52.5%)
	Unknown/not collected	43 (36.4%)
	Hispanic	13 (11.0%)
Primary race (self-reported)	Asian	9 (7.6%)
	White	50 (42.4%)
	African-American	5 (4.2%)
	Other	11 (9.3%)
	Unknown	43 (36.4%)
Sample type	Primary	91 (66.9%)
	Metastasis	28 (20.6%)
	Not collected	1 (0.7%)
	Unspecified	12 (8.8%)
	Not applicable	4 (2.9%)

Within the AACR Project GENIE repository, primary race and ethnicity are defined as distinct, self-reported demographic variables. For somatic mutation frequency analyses and primary vs metastatic tumor comparisons, analysis was performed at the sample level.

### Ethical statement

Institutional Review Board (IRB) approval at Creighton University (Phoenix, AZ, USA) was waived for this study, as it utilized publicly accessible, de-identified data.

### Statistical analysis

To elucidate the functional implications of recurrently mutated genes, Gene Ontology (GO) and Kyoto Encyclopedia of Genes and Genomes (KEGG) pathway enrichment analyses were performed. All statistical analyses were conducted in RStudio (R Foundation for Statistical Computing, Vienna, Austria). Associations between categorical variables, including gene mutations and demographic groups (race, sex), were evaluated using Fisher’s exact or chi-square tests, as appropriate. For subgroup enrichment analyses, all recurrently mutated genes identified in the cohort were assessed across demographic strata. *P*-values were recorded for all associations. Given the large multiple-testing burden and variable gene coverage across samples, we do not interpret nominal *P* values as evidence of statistical significance. Accordingly, results are presented descriptively and framed as hypothesis-generating. Where reported, raw *P* values are included only as descriptive metrics and are not used to support inferential claims.

## Results

### Patient demographics of AE

Patient demographics are summarized in Table 1. This study included 136 samples from 118 patients, of whom 53 (44.9%) were male, 63 (53.4%) were female, and sex was unavailable for 2 (1.7%) patients. By age group, 84 (71.19%) were pediatric cases and 34 (28.81%) were adult cases. Regarding the ethnic composition of our study, 62 (52.5%) patients were Non-Hispanic, 13 (11.0%) were Hispanic/Latino, and 43 (36.4%) had unknown ethnicity. Of the cohort, 50 (42.4%) were White, 9 (7.6%) Asian, 5 (4.2%) African-American, 11 (9.3%) classified as other, and 43 (36.4%) had unknown ethnicity. In this cohort, 91 (66.9%) samples were categorized as primary tumors, 28 (20.6%) as metastatic tumors, 12 (8.8%) as unspecified, 4 (2.9%) as not applicable, and 1 (0.7%) as not collected.

### Most common somatic mutations and CNAs

Figure 1 illustrates the recurrent genomic alterations identified within this AE cohort. Somatic mutation frequencies were calculated using the total cohort size (*n* ═ 136 samples) as the denominator. The most frequently mutated genes included *TERT* (*n* ═ 17; 12.5%), tumor protein p53 (*TP53)* (*n* ═ 13; 9.6%), lysine methyltransferase 2D (*KMT2D)* (*n* ═ 13; 9.6%), protein kinase, DNA-activated, catalytic subunit (*PRKDC)* (*n* ═ 10; 7.4%), *CDKN2A* (*n* ═ 9; 6.6%), adhesion G protein-coupled receptor A2 (*ADGRA2*) (*n* ═ 8; 5.9%), *EP300* (*n* ═ 7; 5.1%), notch receptor 1 (*NOTCH1*) (*n* ═ 7; 5.1%), lysine methyltransferase 2C (KMT2C) (*n* ═ 7; 5.1%), additional sex combs like 1 (*ASXL1*) (*n* ═ 7; 5.1%), CREB binding protein (*CREBBP*) (*n* ═ 6; 4.4%), ATM serine/threonine kinase (*ATM*) (*n* ═ 6; 4.4%), SET domain containing 2, histone lysine methyltransferase (*SETD2*) (*n* ═ 6; 4.4%), notch receptor 2 (*NOTCH2*) (*n* ═ 6; 4.4%), and lysine methyltransferase 2A (*KMT2A*) (*n* ═ 6; 4.4%). The frequent *TERT* alterations observed primarily consist of promoter mutations. Recurrent CNAs were profiled in 52 samples, with notable homozygous deletion events primarily affecting tumor suppressor genes such as cyclin dependent kinase inhibitor 2B (*CDKN2B*) (*n* ═ 6; 11.5%) and *CDKN2A* (*n* ═ 6; 11.5%). Additional homozygous deletions included methylthioadenosine phosphorylase (*MTAP*) (*n* ═ 4; 7.7%), cytokine receptor like factor 2 (*CRLF2*) (*n* ═ 3; 5.8%), interferon regulatory factor 4 (*IRF4*) (*n* ═ 2; 3.8%), and BRCA1 associated protein 1 (*BAP1*) (*n* ═ 2; 3.8%). Amplification events were rare in comparison, with the most common being CREB regulated transcription coactivator 1 (*CRTC1*) (*n* ═ 1; 1.9%). CNA data were derived from 52 samples, representing a subset sequenced on platforms that reported CNAs to cBioPortal. This may bias CNA frequencies towards more comprehensively profiled samples; therefore, frequencies are reported relative to this subset rather than the entire cohort.

**Figure 1.**
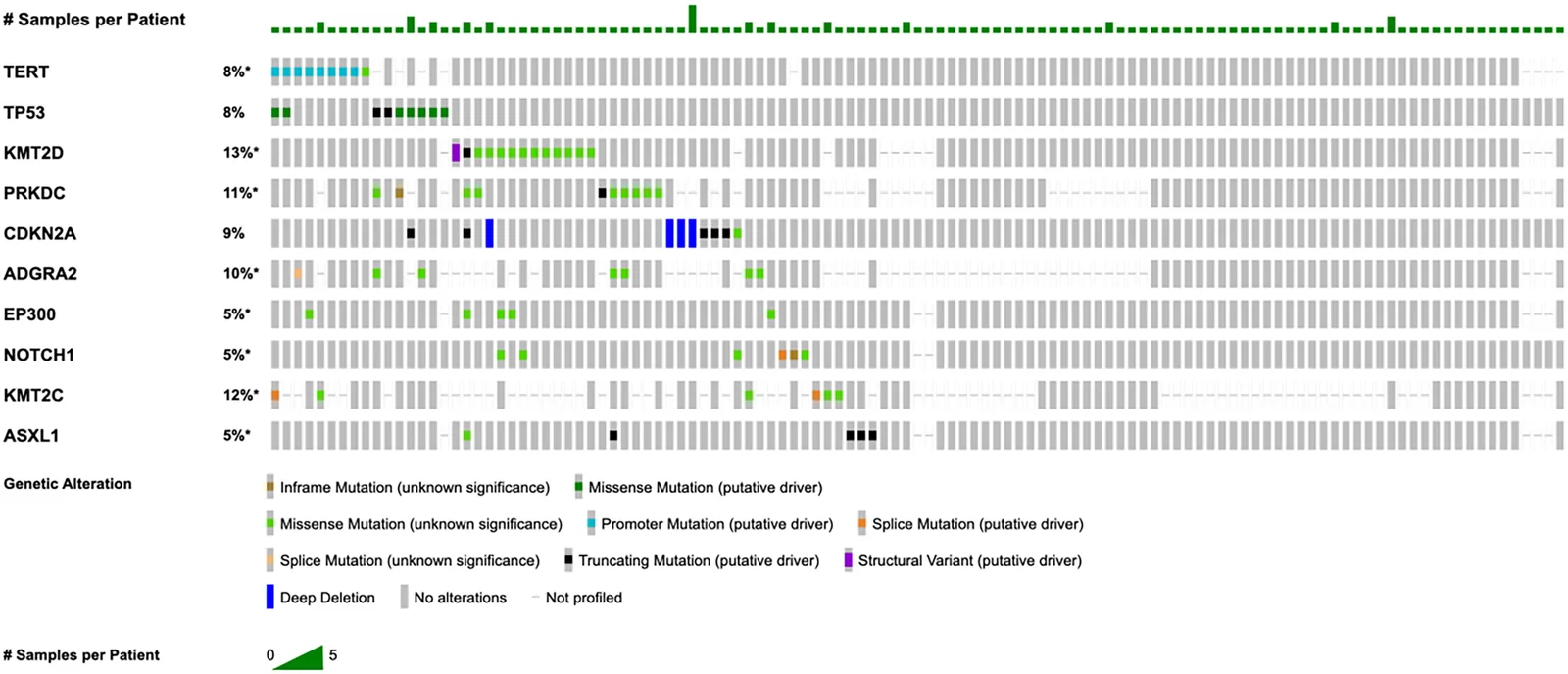
**OncoPrint of recurrent genomic alterations in anaplastic ependymoma.** Columns represent individual tumor samples, and rows represent the 10 most frequently altered genes in the cohort. The upper green bar indicates the number of samples available per patient. Colored boxes denote the type of genomic alteration identified in each sample, including missense, inframe, splice-site, truncating, promoter, and structural variants, as well as deep deletions; light gray indicates no alteration, whereas dark gray indicates that the gene was not profiled. The OncoPrint was generated in cBioPortal and integrates somatic sequence variants with copy-number events. As shown, *TERT* alterations were predominantly promoter mutations, while recurrent deep deletions were most evident in *CDKN2A*. Percentages displayed next to each gene were calculated by cBioPortal as the proportion of profiled samples harboring an alteration in that gene and therefore reflect gene-specific denominators. These values are not directly comparable to the cohort-level frequencies reported in the Results section, which were calculated using all 136 samples; copy-number events additionally reflect the subset of 52 samples with available copy-number data. **Abbreviations:** *CDKN2A*, cyclin dependent kinase inhibitor 2A; *TERT*, telomerase reverse transcriptase.

### Genetic differences by race and sex

Mutation counts were analyzed across race and sex strata to characterize observed variations within the cohort. Due to heterogeneous gene panel coverage among contributing institutions, denominators for demographic comparisons vary by gene. Accordingly, all results and associated *P*-values should be interpreted as descriptive and hypothesis-generating rather than indicative of statistical significance. Among African-American patients, mutations were detected in signal transducer and activator of transcription 6 (*STAT6*) (*n* ═ 1; *P <* 0.001), large tumor suppressor kinase 1 (*LATS1*) (*n* ═ 1; *P <* 0.001), TSC complex subunit 1 (*TSC1)* (*n* ═ 1; *P <* 0.001), forkhead box L2 (*FOXL2)* (*n* ═ 1; *P ═* 0.00172), syntaxin 5 (*STX5)* (*n* ═ 1; *P ═* 0.00250), DOT1 like histone lysine methyltransferase (*DOT1L)* (*n* ═ 1; *P ═* 0.00680), and zinc finger and BTB domain containing 20 (*ZBTB20)* (*n* ═ 1; *P ═* 0.0113). In Asian patients, alterations in catenin alpha 1 (*CTNNA1)* (*n* ═ 1; *P ═* 0.0412) and mitochondrially encoded NADH:ubiquinone oxidoreductase core subunit 5 (*MT-ND5)* (*n* ═ 1; *P ═* 0.0449) were noted. Some alterations were observed in multiple racial subgroups but not across all groups. For instance, ROS proto-oncogene 1, receptor tyrosine kinase (*ROS1)* (*n* ═ 1 vs. *n* ═ 1; *P ═* 0.0104) and EPH receptor B1 (*EPHB1)* (*n* ═ 1 vs. *n* ═ 1; *P ═* 0.0340) were identified in both Asian and African-American patients. Alterations in cyclin dependent kinase inhibitor 1A (*CDKN1A)* (*n* ═ 1 vs. *n* ═ 1; *P ═* 0.0148), *NOTCH2* (*n* ═ 1 vs. *n* ═ 2; *P ═* 0.0236), ABL proto-oncogene 1, non-receptor tyrosine kinase (*ABL1)* (*n* ═ 1 vs. *n* ═ 2; *P ═* 0.0348), AT-rich interaction domain 1A (*ARID1A)* (*n* ═ 1 vs. *n* ═ 1; *P ═* 0.0418), *BRCA1* (*n* ═ 1 vs. *n* ═ 1; *P ═* 0.0418), and polybromo 1 (*PBRM1)* (*n* ═ 1 vs. *n* ═ 1; *P ═* 0.0472) were detected among African-American and White patients. Only thymine DNA glycosylase (*TDG)* (*n* ═ 1 vs. *n* ═ 1; *P ═* 0.0205) was found among both Asian and White patients.

Table 2 presents sex-based comparisons, revealing limited differences. Mutations in *NF2* (*n* ═ 4; *P ═* 0.0433) were identified in male patients but absent in female patients. To avoid overinterpretation of sparse subgroup counts, Table 2 highlights a focused subset of alterations observed across demographic strata, while additional low-frequency observations (*n* ═ 1) are noted in the text but not emphasized in the table.

**Table 2:** Exploratory associations with demographic variables

**Gene**	**Asian, *n* (%)**	**African–American, *n* (%)**	**White, *n* (%)**	***P* value**
*ROS1*	1 (11.11)	1 (25.00)	0 (0.00)	0.0104
*CDKN1A*	0 (0.00)	1 (33.33)	1 (2.22)	0.0148
*TDG*	1 (100.00)	0 (0.00)	1 (10.00)	0.0205
*NOTCH2*	0 (0.00)	1 (50.00)	2 (5.13)	0.0236
*EPHB1*	1 (14.29)	1 (25.00)	0 (0.00)	0.0340
*ABL1*	0 (0.00)	1 (25.00)	1 (2.00)	0.0348
*ARID1A*	0 (0.00)	1 (25.00)	1 (2.13)	0.0418
*BRCA1*	0 (0.00)	1 (25.00)	1 (2.13)	0.0418
*PBRM1*	0 (0.00)	1 (25.00)	1 (2.22)	0.0472
	**Male, *n* (%)**	**Female, *n* (%)**		
*NF2*	4 (7.84)	0 (0.00)		0.0433

Values are presented as *n* (%), where *n* indicates the number of patients within each demographic subgroup exhibiting an alteration in the specified gene. Percentages represent the proportion of patients profiled for that gene within each subgroup, as defined by cBioPortal at the time of analysis, and do not correspond to the total subgroup sizes presented in Table 1. The per-gene subgroup denominators (N) could not be retrospectively retrieved following the retirement of GENIE v18. All *P* values are descriptive, derived from two-sided Fisher’s exact tests, and are reported solely for hypothesis-generating purposes: They should not be interpreted as evidence of statistical significance. **Abbreviations:** *ABL1*, ABL proto-oncogene 1, non-receptor tyrosine kinase; *ARID1A*, AT-rich interaction domain 1A; *BRCA1*, BRCA1 DNA repair associated; *CDKN1A*, cyclin dependent kinase inhibitor 1A; *EPHB1*, EPH receptor B1; GENIE, Genomics Evidence Neoplasia Information Exchange; N, number of profiled patients within each subgroup for a given gene; *NF2*, NF2, moesin-ezrin-radixin like (MERLIN) tumor suppressor; *NOTCH2*, notch receptor 2; n, number of patients with an alteration; *PBRM1*, polybromo 1; *ROS1*, ROS proto-oncogene 1, receptor tyrosine kinase; *TDG*, thymine DNA glycosylase.

### Co-occurrence and mutual exclusivity of mutations

Co-occurrence analyses were conducted for exploratory purposes; *P*-values derived from Fisher’s exact tests are reported descriptively and should not be interpreted as indicative of statistically significant relationships between gene pairs. No patterns of mutual exclusivity were identified; however, several potential co-occurrence patterns emerged among mutated genes. Co-occurrence frequencies for each gene pair are expressed as *n*/*N*, where *N* represents the number of patients with an alteration in the first-listed gene profiled for the second-listed gene, and n denotes the number of those patients with alterations in both genes. Potential co-occurrences were observed between *TP53* mutations and *CREBBP* (*n* ═ 2/5; *P ═* 0.008), *KMT2D* and *EP300* (*n* ═ 2/6; *P ═* 0.011), and *SETD2* and *NOTCH2* (*n* ═ 1/2; *P ═* 0.038), although interpretation is limited by small sample sizes and variable gene panel coverage.

We also examined potential mutation hotspots in the most frequently altered genes. Notably, *TP53* mutations predominantly clustered within the DNA-binding domain and were primarily missense variants (*n* ═ 10/13), as illustrated in the lollipop plot (Figure 2).

**Figure 2.**
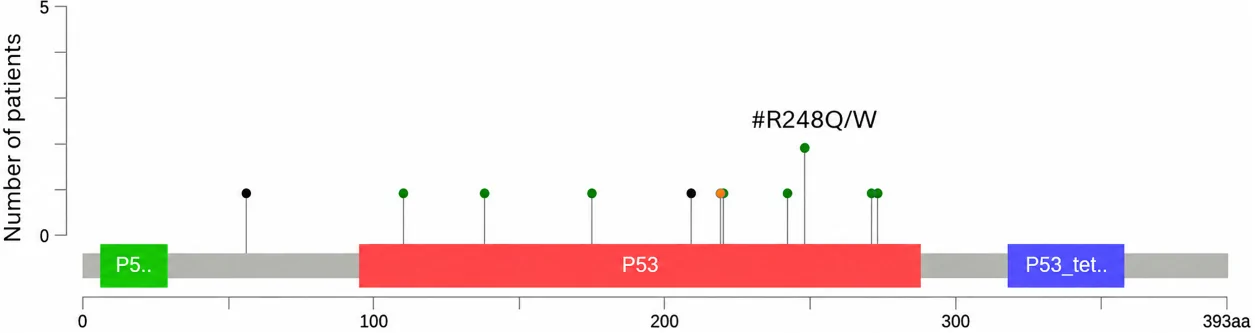
**Lollipop plot of recurrent *TP53* mutation sites in anaplastic ependymoma.** Each lollipop represents an altered amino acid residue in the *TP53*-encoded protein, and lollipop height indicates the number of patients harboring a mutation at that position. Mutations clustered predominantly within the central DNA-binding domain and were mainly missense variants, consistent with the overall *TP53* mutation profile observed in the cohort (10/13). A recurrent hotspot was identified at codon 248 (R248Q/W). The protein schematic below the plot illustrates the major functional domains and aa coordinates of the *TP53*-encoded protein.** Abbreviations:** Aa, amino acid; DNA, deoxyribonucleic acid; *TP53*, tumor protein p53.

### Primary vs. metastatic mutations

Our cohort comprised 91 primary tumors and 28 metastatic cases of AE. The imbalance in sample composition may introduce bias and contribute to variability in observed results. Mutations in O-GlcNAcase (*OGA)* (*n* ═ 2; *P ═* 0.0395) and MutS homolog 3 (*MSH3)* (*n* ═ 2; *P ═* 0.0439) were detected in metastatic tumors but not in primary tumor samples. These findings are descriptive and hypothesis-generating, and should not be construed as statistically significant enrichment due to the limited number of metastatic samples.

### Pathway enrichment analysis

To elucidate the functional significance of the most frequently mutated genes, we conducted a pathway enrichment analysis. This analysis indicated enrichment in genes associated with chromatin modification (GO:0016568), covalent chromatin modification (GO:0016569), and regulation of the cell cycle (GO:0051726), suggesting that these processes may be recurrently affected in AE. Pathway enrichment results are presented to provide biological context for recurrent alterations and are not intended to imply statistically validated pathway-level associations. Collectively, these findings describe recurrent genomic alterations and exploratory demographic and co-occurrence patterns within a histologically defined AE cohort, without suggesting statistically significant associations. The pathway-level observations aim to provide biological context for recurrent alterations and should not be interpreted as validated enrichment signals.

## Discussion

### Subgroups and mutational landscape

The primary objective of this study was to characterize the mutational landscape and disrupted pathways defining histologically defined AE using the AACR Project GENIE repository. Our analysis explored alteration patterns stratified by sex, race, and sample type; these observations are descriptive and hypothesis-generating given the small subgroup sizes and heterogeneous gene coverage.

The majority of the AE cohort consisted of pediatric patients (*n* ═ 84, 71.19%), reflecting established patterns in AE, where children exhibit a higher incidence [9, 10]. Sex-stratification analysis revealed a male-to-female distribution of 44.9% to 53.4%, consistent with prior literature [11]. Mutations in *NF2*, which encodes a tumor suppressor protein regulating cell proliferation, adhesion, and cytoskeletal organization, were observed in male patients [14]. Further research is necessary to understand how certain mutations differ by sex and their impact on patient outcomes.

While our study indicated that White individuals had the highest incidence rate (*n* ═ 50; 42.4%), previous studies using the Surveillance, Epidemiology, and End Results (SEER) cancer database report AE as more frequent in the African-American community [2]. Our study also identified genetic profile differences between White, Asian, and African-American patients within our cohort. Mutations in genes such as *STAT6* (*n* ═ 1; *P <* 0.01), *LATS1* (*n* ═ 1; *P <* 0.01), *TSC1* (*n* ═ 1; *P <* 0.01), *FOXL2* (*n* ═ 1; *P <* 0.01), *STX5* (*n* ═ 1; *P <* 0.01), *DOT1L* (*n* ═ 1; *P <* 0.01), and *ZBTB20* (*n* ═ 1; *P <* 0.0113) were observed in African-American patients. An article by Donson et al. [15] noted that non-recurrent ependymoma patients exhibited increased levels of STAT6 expression, suggesting that STAT6, related to immune functions, may correlate with a better prognosis for ependymoma. Genes *CTNNA1* (*n* ═ 1; *P ═* 0.0412) and *MT-ND5* (*n* ═ 1; *P ═* 0.0449) were identified in single Asian patients each. Genes *ROS1* and *EPHB1* (*n* ═ 1 vs *n* ═ 1; *P ═* 0.0104) were found in both an Asian and an African-American patient. Mutations in *CDKN1A* (*n* ═ 1 vs *n* ═ 1; *P ═* 0.0148), *NOTCH2* (*n* ═ 1 vs *n* ═ 2; *P ═* 0.0236), *ABL1* (*n* ═ 1 vs *n* ═ 2; *P ═* 0.0348), *ARID1A* (*n* ═ 1 vs *n* ═ 1; *P ═* 0.0418), *BRCA1* (*n* ═ 1 vs *n* ═ 1; *P ═* 0.0418), and *PBRM1* (*n* ═ 1 vs *n* ═ 1; *P ═* 0.0472) were identified among both African-American and White patients. The gene *BRCA1* has been linked to ethnic treatment disparities in other cancer types. A study by Bhaskaran et al. [16] involving over 30,000 Chinese patients diagnosed with ovarian and breast cancers found that treatment strategies for BRCA-related cancers are often inadequate for non-Caucasian populations. Only one gene, *TDG* (*n* ═ 1 vs *n* ═ 1; *P ═* 0.0205), was mutated in both African-American and White patients. These subgroup observations should be interpreted with caution and warrant validation in larger, molecularly defined cohorts with consistent genomic profiling.

### Commonly mutated genes and known pathways

Genomic profiling of our AE cohort revealed a complex mutational landscape characterized by alterations in epigenetic regulators, the NOTCH signaling pathway, and cell cycle genes. The most frequently mutated genes included *TERT* promoter mutations (*n* ═ 17; 12.5%), followed by the tumor suppressor *TP53* (9.6%). Mutations in chromatin remodeling machinery were prevalent, with alterations observed in *KMT2D* (9.6%), *KMT2A* (4.4%), *KMT2C* (5.1%), *EP300* (5%), *CREBBP* (4.4%), *ASXL1* (5.1%), and *SETD2* (4.4%). DNA repair pathways also exhibited mutations in *PRKDC* (7.4%) and *ATM* (4.4%), while alterations in *CDKN2A* (6.6%), *NOTCH1* (5.1%), and *NOTCH2* (4.4%) impacted cell cycle and developmental signaling pathways. Additional mutations were identified in *ADGRA2* (5.9%), and tumor suppressor genes such as *MTAP* (7.7%), *CRLF2* (5.8%), *IRF4* (3.8%), and *BAP1* (3.8%).

Somatic mutations within our AE cohort ranged from epigenetic modifications to alterations in the NOTCH signaling pathway, cell cycle, and chromatin remodeling pathways. In terms of treatment strategies for ependymoma, gross total resection followed by postoperative adjuvant radiotherapy remains the standard of care for AE [17]. However, no prospective dose-response studies have been conducted examining radiotherapy in AE patients [18, 19]. Prior literature has established that damage response pathways within cancer cells can promote radiotherapy resistance [20]. Mutations in genes associated with DNA damage response and cell cycle pathways may lead to radiosensitivity in AE patients.

### TERT mutations

Previous studies on AE have extensively investigated the *TERT* gene [21–24]. Mutations within the *TERT* promoter region have been documented in various cancers. *TERT* promoter mutations are traditionally associated with poorer progression-free survival [25]. Within our AE cohort, *TERT* promoter mutations (12.5%) were the most frequently observed alterations.

Reactivation of *TERT* facilitates the proliferation of cancer cells and contributes to tumorigenesis. Previous research has indicated that *TERT* serves as a potential prognostic marker for multiple cancers [26]. We propose that *TERT* represents a promising target for therapeutic intervention in AE. Studies have shown that patients with *TERT* promoter mutations tend to be older [25]. In other cancer types, *TERT* promoter mutations are associated with older patients [22, 27]. A study by Koelsche et al. [22] found that in a sample of 48 patients diagnosed with AE, with 28 pediatric patients and 20 adult patients, *TERT* mutations were observed in the adult patients and were not detected in pediatric patients. The presence of *TERT* mutations in pediatric patients within our cohort suggests that *TERT* mutations can appear in younger patients with AE. We suggest that further research should investigate *TERT* inhibitors as an avenue of treatment for AE [28].

### Chromatin remodeling pathway

Chromatin remodeling complexes have been extensively studied in cancer biology. Through histone modifications, these complexes regulate gene expression and facilitate normal cellular development and differentiation. Mutations in key genes disrupt histone modifications, hindering cellular differentiation and impairing tumor suppressor functions. In our AE cohort, the most frequent alterations involved chromatin remodeling pathways, particularly the complex of proteins associated with Set1 (COMPASS) complex, the CREB-binding protein (CBP) and p300 (EP300) complex, and the Polycomb repressive complex. These alterations were primarily driven by mutations in *KMT2D* (9%), *KMT2A* (4.5%), and *KMT2C* (5%) within the COMPASS complex; [29] *EP300* (5%) and *CREBBP* (4.5%) within the CBP/p300 complex; and *ASXL1* (5%) within the Polycomb repressive complex.

### NOTCH signaling pathway

The NOTCH signaling pathway has been well-documented in the literature within AE [30, 31]. In a pediatric patient diagnosed with AE, a *NOTCH1* germline mutation was identified, implicating dysregulation of the NOTCH signaling pathway. Dysregulation of this pathway [32] has been suggested as a factor in ependymoma tumorigenesis. Within our cohort, *NOTCH1* (5.1%), *NOTCH2* (4.4%), *EP300* (5%), and *CREBBP* (4.4%) were among the most frequently mutated genes. *CREBBP* and *EP300* have been shown to indirectly upregulate and function as co-activators in the NOTCH signaling pathway [33, 34]. Given the recurrent alterations in genes associated with the NOTCH pathway, we propose that inhibitors of this pathway, such as γ-Secretase, may warrant further investigation as potential therapeutic strategies for AE [35].

### Cell cycle

Dysfunctional genes integral to the cell cycle have been widely implicated in tumorigenesis. Our analysis revealed frequent alterations in the genes *TP53* (9.6%), *ATM* (4.4%), and *CDKN2A* (6.6%), suggesting impaired cell cycle control in AE. Pathway analysis indicated that mutations in *ATM* and *TP53* may adversely affect the DNA damage response by impairing the p53 activation pathway. Specifically, *ATM* mutations can prevent the release of the tumor suppressor p53 following DNA damage, inhibiting apoptosis [36].

### Pathway analysis reveals disruption of core cellular processes

Our pathway enrichment analysis provided mechanistic insights into the observed mutational landscape. The enrichment of mutations in genes related to chromatin remodeling and histone modification (e.g., *KMT2D, EP300, CREBBP, ASXL1*) aligns with a growing body of evidence suggesting that epigenetic dysregulation is a central oncogenic driver in many CNS tumors. These alterations can lead to significant changes in gene expression, promoting cell proliferation and blocking differentiation [37, 38]. Similarly, recurrent mutations affecting NOTCH and p53 signaling pathway genes are consistent with their established roles in ependymoma tumorigenesis [39, 40]. This convergence of mutations on a limited number of core pathways suggests that, despite genomic heterogeneity, these tumors may share common dependencies that could be targeted therapeutically. Given the absence of clinical outcome data, the potential therapeutic relevance of these pathways should be regarded as hypothetical.

### Co-occurrence patterns and functional implications

We identified co-occurrence patterns involving the genes *TP53* and *CREBBP*, *KMT2D* and *EP300*, and *SETD2* and *NOTCH2*. Prior studies [41] in other cancer types have suggested potential prognostic relevance of *TP53*–*CREBBP* co-alteration; however, no survival or outcome data were available in this cohort. *TP53* and *CREBBP* co-occurred in a small subset of cases (*n* ═ 2/5; *P <* 0.01), consistent with previous reports indicating potential outcome relevance in other malignancies [4]. These genes participate in chromatin remodeling and transcriptional regulation pathways, suggesting potential cooperative epigenetic dysregulation in AE. For instance, *CREBBP* and *EP300* encode histone acetyltransferases that regulate gene expression, while *TP53* and *KMT2D* function in tumor suppression and chromatin modification pathways. The concurrent alteration of these genes may indicate a coordinated disruption of chromatin structure and cell cycle regulation. However, as the AACR GENIE dataset lacks survival or outcome data for this cohort, the clinical or prognostic significance of these co-occurring mutations remains undetermined and should be considered hypothesis-generating.

### Comparisons between primary and metastatic samples

Our analysis revealed significant findings through the comparison of primary and metastatic AE samples. Mutations in *OGA* (*n* ═ 2; *P ═* 0.0395) and *MSH3* (*n* ═ 2; *P ═* 0.0439) were exclusively observed in metastatic tumors. Similar findings in colorectal cancer have reported associations between *MSH3* dysregulation and advanced disease stages [42]; however, these observations should be considered in biological context and not interpreted as evidence of shared clinical behavior in AE. The enrichment of *MSH3* mutations in metastases suggests it may warrant further investigation in relation to tumor progression.

### Limitations

#### Histopathologically-defined cohort limitations

Our study presents several limitations. Primarily, it relies on a historical cohort defined by the histological diagnosis of “anaplastic ependymoma” rather than the molecularly defined entities outlined in the 2021 WHO classification. This historical designation encompasses multiple molecularly distinct entities according to current WHO criteria. The publicly accessible AACR Project GENIE repository does not include the DNA methylation and RNA sequencing data required to reclassify these tumors into contemporary, clinically relevant subgroups, such as supratentorial ZFTA fusion-positive tumors or Posterior Fossa Group A (PFA) ependymomas. This heterogeneity within our histologically defined cohort may obscure subtype-specific mutational patterns, representing a significant limitation. Therefore, the results of this study should not be interpreted as evidence of common oncogenic mechanisms across all ependymoma subtypes.

#### Statistical power limitations

Secondly, our sample size of 118 patients, although one of the largest genomic cohorts for this historically defined rare tumor, limits the statistical power to detect infrequent mutations and robustly associate mutations with demographic features. Several nominal findings related to sex and race were driven by one or two alterations within small subgroups, further constraining statistical power and precision. Given the statistical imbalance and limited sample size, findings from comparisons between primary and metastatic tumor samples, as well as those from racial and sex-based analyses, should be regarded as exploratory, race-specific, and hypothesis-generating, with wide confidence intervals. Validation in larger, prospectively collected, and molecularly stratified cohorts is essential to confirm these observations.

#### Demographic analysis limitations

Thirdly, the AACR Project GENIE repository aggregates data generated using heterogeneous sequencing panels, leading to variable gene coverage across samples. Consequently, denominators for demographic enrichment analyses differ by gene and subgroup, as percentages reflect the proportion of patients profiled for and altered in a given gene rather than total subgroup sizes. This variability limits direct comparison across demographic groups and necessitates that enrichment findings be interpreted as exploratory.

#### Lack of patient outcome data

Fourthly, the publicly available GENIE repository does not provide patient outcome data (e.g., progression-free or overall survival) for this cohort. Consequently, we were unable to conduct survival analyses or formally establish the prognostic significance of the identified mutations. Addressing the therapeutic or prognostic relevance of our findings would require integration with independent, publicly available datasets concerning AE samples, which was beyond the scope of this study. All interpretations regarding the potential impact of these genes are based on their known biological functions and published literature, rather than clinical outcomes from our cohort.

#### Lack of RNA-Seq data

Fifth, the dataset lacks transcriptomic (RNA-Seq) data, preventing analysis of the tumor immune microenvironment. Computational deconvolution algorithms, such as Cell-type Identification By Estimating Relative Subsets Of RNA Transcripts (CIBERSORT) and xCell, utilize gene expression data to assess immune cell infiltration. Therefore, claims about the immunologic landscape of AE cannot be substantiated by this study.

#### Lack of longitudinally collected samples

Sixth, without longitudinally collected samples from individual patients across different stages of tumor progression, the study could not distinguish mutations with functional relevance from incidental alterations.

#### Presence of dependent samples

Seventh, a potential source of bias arises from the presence of dependent samples within our dataset, such as multiple tumor specimens from the same patient. To mitigate this dependency, demographic enrichment analyses were conducted at the patient level.

#### Lack of treatment history and clinical data

Eighth, the analysis of treatment effectiveness across mutational profiles and histologic subtypes was hindered by the database’s lack of treatment history and clinical intervention records. This limitation restricts our ability to account for intervention-induced mutations that could influence evolutionary analyses of AE.

#### Methodology constraints

Ninth, the repository comprises data collected from multiple centers and institutions, each employing varying intra-institutional sequencing protocols and technologies. These differences could have impacted how mutations were detected and quantified. Consequently, our estimated mutation rates may not accurately reflect true mutation rates. Recognizing these constraints, our study contributes to the characterization of the genomic profile of AE and identifies potential new targets for therapeutic intervention.

#### Additional limitations

Additionally, due to the AACR Project GENIE’s transition to v19, age stratification analysis by pediatric and adult subgroups could not be retrospectively performed following our initial analysis of v18. Specific false discovery rate (FDR)-corrected q-values from the original v18 analysis were not recorded and cannot be obtained retrospectively due to access constraints following cBioPortal’s transition to v19. Co-occurrence analyses were based on Fisher’s exact test *P*-values without calculation of odds ratios or confidence intervals. Given the small sample sizes and sparse contingency tables, these findings should be considered exploratory and require validation in larger cohorts.

Finally, this investigation is entirely computational and should be regarded as hypothesis-generating. The translational value of our findings remains speculative until validated in independent, molecularly classified patient cohorts and through functional wet-lab experiments. Such studies, including proteomic analyses, functional perturbations in cell lines, and drug-response assays, are critical next steps to confirm the biological roles of these mutations and evaluate their potential as therapeutic targets.

## Conclusion

This study, utilizing the AACR GENIE repository, characterized the genomic and demographic profiles of patients diagnosed with AE, revealing alterations in chromatin remodeling, NOTCH signaling, and cell cycle control genes. Somatic mutations in *TERT*, *TP53*, *KMT2D*, *KMT2A*, *KMT2C*, *EP300*, *CREBBP*, *ASXL1*, *SETD2*, *PRKDC*, *ATM*, *CDKN2A*, *NOTCH1*, *NOTCH2*, and *ADGRA2* underscore the need for further investigation into how mutations within these known pathways (NOTCH signaling pathways, ATM-p53 pathway) affect treatment response [12, 13]. *TERT* promoter mutations emerged as the most frequently mutated, appearing in both pediatric and adult patients, while metastatic tumors showed enrichment of *OGA* and *MSH3*. Our analysis also revealed novel insights, including the observation of *TERT* promoter mutations in pediatric AE patients, which contrasts with prior literature reporting *TERT* mutations only in adults.

Our study delineates the genomic landscape of AE and identifies exploratory race- and sex-stratified patterns within this cohort (*STAT6* in African-American patients and *BRCA1* in both African-American and White patients; *NF2* in males).

We propose the involvement of candidate pathways that may warrant further investigation for therapeutic relevance in AE: *TERT* inhibitors and NOTCH pathway inhibitors. Future investigations with larger, molecularly defined cohorts and integrated clinical outcome data could yield crucial insights into the mechanisms underpinning AE biology. Our findings provide an initial framework and rationale for future experimental studies and validation in independent clinical cohorts.

## Data Availability

The data presented in this study are available from the AACR GENIE Database at https://genie.cbioportal.org/ (accessed on 27 July 2025).
